# Psychosocial distress in acute cancer patients assessed with an expert rating scale

**DOI:** 10.1007/s00520-010-0850-9

**Published:** 2010-04-11

**Authors:** Bianca Senf, Holger Brandt, Axel Dignass, Rolf Kleinschmidt, Jochen Kaiser

**Affiliations:** 1 Medical Clinic, Markus Hospital, Wilhelm-Epstein-Str. 2, 60431 Frankfurt am Main, Germany; 2 Institute of Medical Psychology, Goethe University, 60528 Frankfurt am Main, Germany; 3 Institute of Psychology, Goethe University, 60054 Frankfurt am Main, Germany

**Keywords:** Cancer, Psychosocial distress, Expert rating, Sociodemographic predictors, Disease-related predictors

## Abstract

**Purpose:**

The identification of psychosocial stress in cancer patients has remained a challenging task especially in an acute care environment. The aims of the present study were to apply a short expert rating scale for the assessment of distress during the acute treatment phase and to identify potential sociodemographic and disease-related predictors.

**Methods:**

Four hundred seventy-eight ward cancer patients were assessed with the short form of the psycho-oncological basis documentation and its breast-cancer-specific version. In addition, they completed a self-rating questionnaire on stress in cancer patients. We recorded sociodemographic and disease-related variables and assessed their predictive value for psychosocial distress.

**Results:**

According to the expert rating scale, 56.3% of patients were rated distressed. While only 31.3% of patients were classified as distressed according to a patient self-rating, both approaches showed a good degree of concurrence with a consistent classification of 69% of patients. Younger age, current psychotropic medication, and past psychological treatment were associated with higher distress levels. Patients with metastases and those with a poorer functional status were more distressed. Interestingly, having an operation was associated with a better psychological well-being.

**Conclusions:**

This study demonstrated that a substantial proportion of cancer patients in acute care are psychosocially distressed. A short expert rating scale proved to be a feasible tool for the assessment of distress in an acute care setting.

## Introduction

Cancer patients suffer not only from physical symptoms but also from the psychological and social stress associated with both diagnosis and treatment of their disease [1]. Apart from the fear of dying, patients feel threatened by interventions like chemo- or radiotherapy, and they worry about losing their bodily integrity, independence, and social roles [2, 3]. Sleep disturbances, restlessness, and diffuse anxiety are frequently reported symptoms. According to current estimations, about 25–45% of cancer patients show clinically relevant levels of anxiety and depression [4, 5]. The effectiveness of psychosocial interventions for cancer patients is still a matter of debate [6–9]. On the basis of a methodologically rigorous meta-analysis, Newell et al. [10] have tentatively recommended some interventions including cognitive–behavioral therapy, counseling, and hypnosis for the treatment of symptoms like anxiety and general affect and for the enhancement of coping skills and quality of life. However, further methodologically sound trials are needed to establish the effectiveness of therapeutic strategies.

Unfortunately, psychological distress may remain unrecognized in some cancer patients. Patients either do not communicate their feelings or they may be unaware of their distress [11]. On the other hand, estimations by doctors or nurses have only limited accuracy [12]. Thus, although untreated psychosocial stress can negatively influence both the patients' quality of life, their ability to cope with the disease, and the outcome of medical interventions, many patients may not obtain the psycho-oncological treatment they need [12, 13]. This may be particularly true for patients in acute care settings. Here, only few studies have investigated the need for psycho-oncological support [14, 15].

A key problem in acute care is the economic assessment of the subjective well-being of cancer patients. Previous approaches have used self-rating questionnaires designed to assess emotional disorders [5, 16, 17]. Self-rating scales developed in a psychiatric context, however, are not entirely appropriate for cancer patients [3]. For example, adequate fears may be interpreted as signs of a mental disorder, whereas specific cancer-related problems are not addressed. Moreover, distress below psychiatric levels may already require psycho-oncological treatment. Alternatively, self-rating questionnaires [18, 19] and a distress thermometer [20] have been devised to measure cancer-specific distress. However, their validity may be affected by denial, which in some patients may play a role as a coping strategy protecting them against overwhelming experiences and the associated feelings [21]. To overcome these limitations, the basic documentation for psycho-oncology (PO-Bado) has been developed as a cancer-specific expert rating scale [22, 23]. As this scale still turned out to be too long for integration in the routine admission procedure in acute clinical settings, a six-item short form (basic documentation for psycho-oncology short form (PO-Bado SF)) was devised [24].

The first aim of the present study was to apply the short, cancer-specific expert rating instrument PO-Bado SF to assess the distress levels of cancer patients in acute care. To this end, a routine screening of all patients admitted within a 6-month period was conducted in an acute hospital. Since external patient assessments and self-ratings often do not concur, the PO-Bado SF was compared with the self-assessment Questionnaire on Stress in Cancer Patients (QSC-R23, [18]). The second aim was to assess possible influences of sociodemographic and disease-related variables on psychosocial distress.

## Materials and methods

### Participants

Five hundred seventy ward cancer patients treated over a 6-month period at the “Frankfurter Diakoniekliniken” (Markus Hospital) in Frankfurt am Main, Germany, were invited to participate in the study. Markus Hospital is a general acute hospital with more than 550 beds. In addition, it serves as a teaching hospital for Goethe University of Frankfurt. It provides the full range of adult clinical specialties, including general, trauma, and plastic surgery, orthopedics, internal medicine, obstetrics/gynecology, urology, psychiatry, and geriatrics. Each year, 1,000–1,200 cancer patients are treated at the Markus Hospital. All patients were either newly admitted with the diagnosis of cancer, or cancer was diagnosed during their hospital stay. All patients had been informed about their cancer diagnosis by a physician prior to testing. Fifty-three patients could not be included due to organizational problems, five patients refused to participate, and 34 did not meet one or more of the following inclusion criteria: minimum age of 18 years, sufficient German language skills, absence of cognitive impairments, and absence of pain or nausea at the time of testing. We considered it unethical to interview patients suffering from strong acute pain or other acute physical ailments like, e.g., nausea or vomiting. Here, priority was given to an immediate treatment of physical symptoms and related psychological problems. It was decided to avoid putting the patients under additional stress by participating in our investigation. This applied to only 34 patients who either received advanced palliative treatment or were recovering from surgery. Thus, data from 478 patients were included in the study. All participants gave their informed written consent. The study was performed in accordance with the ethical standards laid down in the Declaration of Helsinki.

The mean age of the 478 participants (66.7% women) was 63 years (range, 18–95 years). Details about gender, partnership, employment status, diagnoses, disease stage, metastases, and treatment during the 2 months prior to testing are given in Table 1. More than half of the patients (54.4%) were affected by additional somatic disorders. One quarter of the participants (25.1%) reported receiving psychological treatment in the past, and 41.1% had taken psychotropic medication during the last 3 days prior to assessment.

**Table 1:** Sample description

	Samples	Percentage
Gender		
Female	319	66.7
Male	159	33.3
Partnership		
With partner	339	71.4
Without partner	136	28.6
Employment status		
Employed	79	16.8
Retired	260	55.2
Sick leave	77	16.3
Other	55	11.7
Cancer diagnosis		
Breast	219	46.3
Gastrointestinal	84	17.8
Genito-urinary	83	17.6
Other	87	18.4
Disease status		
First occurrence	383	82.2
Secondary tumor	41	8.8
Cancer recurrence	36	7.7
Metastases		
Yes	109	24.1
No	177	39.2
Incomplete staging	166	36.7
Treatment (2 preceding months)		
None	127	27.0
Operation	189	40.2
Chemotherapy	87	18.5
Operation + chemotherapy	30	6.4
Other	37	7.9

### Measures

#### Basic documentation for psycho-oncology short form

The PO-Bado SF [24] includes three parts: questions on demographic and medical characteristics and on psychosocial stress, a manual explaining the rating criteria for the items, and an interview guideline. Materials and information can be downloaded from http://www.po-bado.med.tum.de. A recent evaluation study in 596 cancer patients [24] showed satisfactory internal consistency (Cronbach's alpha, 0.82), inter-rater reliability (three raters, intraclass correlation coefficients between 0.74 and 0.93), and convergent validity with the self-rating scales QSC-R23 [18] and the Hospital Anxiety and Depression Scale [25]. The PO-Bado assesses psychosocial stress within the last 3 days. The interview takes about 10 min. The questions refer to the subjective cancer-related experience of the patient rather than to the intensity of the symptoms. The scale comprises the following six items that are rated on a scale ranging from 0 (not at all) to 4 (very much): (1) fatigue/tiredness, (2) functional limitations in daily activities, (3) mood swings/vulnerability/helplessness, (4) anxiety/worries/tension, (5) depression/grief, and (6) other stressful factors (social, family-related, etc.). The first two items cover the dimension of physical distress, while the remaining four items belong to the psychological stress dimension. At the end, the interviewer judges the patient's overall distress level. High distress would suggest that a patient may need psycho-oncological support.

#### Basic documentation for psycho-oncology breast cancer

The basic documentation for psycho-oncology breast cancer (PO-Bado BC) [24] was developed following requests from clinical staff in breast cancer centers. Compared with the PO-Bado SF, it includes four additional items in the physical distress dimension and six additional items in the psychological stress dimension that are specific to breast cancer. Moreover, the breast cancer version includes questions about “mood swings/uncertainty” and “helplessness/vulnerability” as two separate items, whereas they form a single item in the PO-Bado SF. These two items were combined by averaging in the PO-Bado BC to enable calculation of a common score across the entire sample. While all these items are rated on a scale ranging from 0 (not at all) to 4 (very much) similarly to the PO-Bado SF, the topic of “additional stressful factors” is subdivided into four individual items in the PO-Bado BC, which are rated in a binary manner (0 = yes, 1 = no).

#### Questionnaire on stress in cancer patients

The QSC-R23 [18] is a psychometric self-rating scale for the assessment of psychosocial stress in cancer patients that comprises five subscales: physical performance, (psycho)somatic complaints, anxiety, information deficits, and social problems. The 23 items are rated on six-point Likert scales ranging from 0 (does not apply) to 5 (applies and troubles me a lot). A total score can be calculated by averaging the scores across the single items. To determine high distress levels suggesting a need for psycho-oncological support, cutoff criteria were proposed by the authors [26].

### Procedure

Interviews and ratings with the PO-Bado SF/BC were conducted by psycho-oncologists (i.e., chartered psychotherapists with university degrees in psychology and specialty training in psycho-oncology) and psychology students in their third to fifth year of study. Students received the following intensive training. During an introductory phase, they acquired general knowledge about clinical routines at the oncology wards. They also watched the trained psychologists conduct psycho-oncological interviews (live and on video) and were trained to rate the patients' distress levels. Interviews with the expert rating scale were trained during several role plays. During the first three patient interviews, the students were supervised by a psycho-oncologist.

The interviews took place within the first 3 days of the patient's hospital stay. Prior to each interview, the treating physician was asked about the patient's health status and his/her level of information about the diagnosis. Sociodemographic and medical history data were copied from the patient's medical record. The patient was then informed verbally and in writing about the study and asked to give his/her written consent. Unless the patient was confined to bed, the interview took place in a separate room to protect his/her privacy. The interview was conducted according to the PO-Bado guidelines and manual, including instructions for a structured assessment, detailed definitions of each item and criteria for the item ratings, and sample questions for each item. Breast cancer patients (except for 16 patients who were interviewed with the PO-Bado SF) were interviewed with the PO-Bado BC and all remaining patients with the PO-Bado SF. After the interview, the patient was informed about psycho-oncological treatments offered at the hospital. Finally, he/she was given a copy of the QSC-R23 and asked to complete it by the evening of the same day.

### Data analysis

Only those scales of the PO-Bado SF and BC were analyzed that were identical to both versions of this instrument (for simplicity, we will therefore use the term “PO-Bado SF” when presenting findings obtained on the basis of these common items). An exception was made for the four items of the breast cancer version that refer to different additional stressful factors and are rated in a binary way. Differences between patients with and without indication for psychological intervention were compared on the PO-Bado SF items and the items referring to “other stressful factors” of PO-Bado SF and PO-Bado BC. The expert rating scale PO-Bado SF was compared with the self-rating questionnaire QSC-R23 by determining the proportion of patients for whom both instruments yielded convergent versus divergent classifications. Patients with high distress levels as determined by the PO-Bado SF and participants with low distress levels were compared also on sociodemographic and disease-related variables. Possible differences were tested with *t* tests for the continuous variable age and with *χ* ^2^ tests for all discrete variables. Finally, a multiple logarithmic regression was applied to the two groups in order to determine the combined predictive value of the previously identified significant predictors for high distress.

## Results

### PO-Bado and psychosocial distress

The scores (means and standard deviations) on the items of the PO-Bado SF obtained in the entire patient sample are given in Table 2. An exploratory principal factor analysis (PCA with oblimin rotation) on the five items common to PO-Bado SF and BC showed that two factors explained 76% of the variance: a “physical distress” factor with the most pronounced loadings from the first two items and a “psychological distress” factor with loadings from the three subsequent items; see Table 3. Both factors were moderately correlated (*r* = 0.40). This factor structure corresponded to previous results for the standard PO-Bado [23]. A comparison of average scores for both factors showed higher ratings for psychological distress than for physical complaints (psychological, 1.78 (SD = 0.93); physical, 1.20 (SD = 1.01), *t*(476) = 12.15, *p* < 0.001).

**Table 2:** Means and standard deviations (SD) for the five items, which are included in both PO-Bado SF and BC and for the item “additional emotional problems” included in the SF only

Item	Samples	Mean	SD
Fatigue/tiredness	475	1.28	1.12
Functional limitations in daily activities	472	1.13	1.16
Mood swings/vulnerability/helplessness	469	1.50	1.07
Anxiety/worries/tension	471	2.13	1.03
Depression/grief	474	1.69	1.16
Additional emotional problems	271	1.39	1.26

**Table 3:** Factor loadings for the two PO-Bado SF factors psychological distress and physical distress

	Factor 1: psychological distress	Factor 2: physical distress
Fatigue/tiredness	0.08	0.84
Functional limitations in daily activities	−0.06	0.92
Mood swings/vulnerability/helplessness	0.73	0.18
Anxiety/worries/tension	0.92	-0.10
Depression/grief	0.88	0.00

**Fig. 1:**
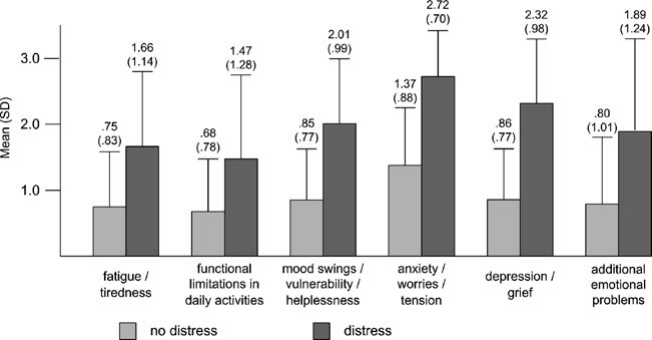
Means and standard deviations (in parentheses) for the PO-Bado items common to both short form (SF) and breast cancer (BC) version as a function of distress ratings. Distressed patients scored higher on each of these factors (*p* < 0.001)

Two hundred fifty-nine patients (56.3%) were identified with high psychosocial distress levels suggesting a potential need for psycho-oncological treatment, whereas 201 patients (43.7%) showed low distress. Missing data precluded a decision about the distress level in 18 patients. Patients with high distress showed significantly higher values for all individual physical and psychological distress items (*p* < 0.001); see Fig. 1. For the PO-Bado BC where additional stressful factors were assessed by four separate items with a binary answer mode, significant associations were revealed between high distress and the presence of each of the additional stress factors by *χ* ^2^ tests (*p* < 0.01).

### Comparison between expert rating and patient self-rating

As distress self-ratings by patients and ratings by external observers may differ, we compared the PO-Bado SF with the results of the QSC-R23 in *N* = 377 patients with complete data on both instruments. Missing data in *N* = 85 patients did not allow to obtain an indication with the QSC. Another 15 patients had not received an indication with the PO-Bado SF; 118 patients (31.3%) were classified as highly distressed with the QSC-R23. There was a high degree of overlap between PO-Bado SF and QSC concerning distress. More than two thirds of patients (68.7%) were classified consistently by both scales. High distress was indicated by PO-Bado SF but not by QSC in over a quarter of the participants (27.6%), whereas the opposite pattern (high distress on QSC but not PO-Bado SF) was found for only 3.7% of patients. The association between both instruments was statistically significant (*χ* ^2^ = 75.46, *df* = 1, *p* < 0.001).

### Sociodemographic variables and psychosocial distress

The only sociodemographic variable that differed between patients with and without high distress levels determined with the PO-Bado SF was age. Distressed patients were on average about 3 years younger than nondistressed participants (mean for distressed patients, 61.3 (SD = 12.3) years; mean for nondistressed patients, 64.2 (SD = 12.3) years, *t*(458) = 2.52, *p* = 0.012). There were no differences between both groups of patients in gender, personal status (partnership, presence of children, and number of children), or employment situation.

### Disease-related variables and psychosocial distress

**Fig. 2:**
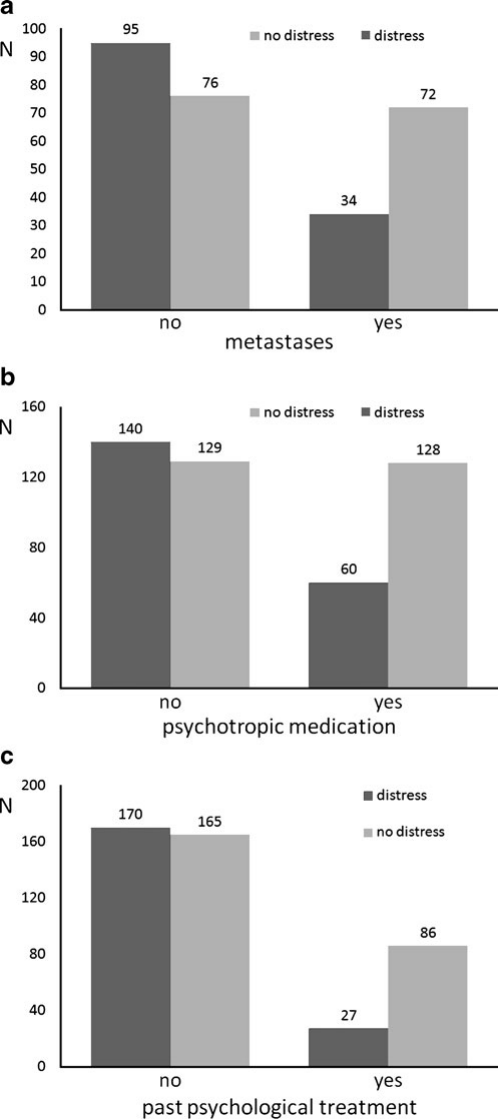
Absolute numbers of patients with high vs. low distress levels as rated with the PO-Bado SF are given as a function of presence of **a** metastases, **b** psychotropic medication, and **c** past psychological treatment (all of these factors were significantly related to PO-Bado distress ratings, see the “Results” section for details)

Psychosocial distress did not differ between patients with different cancer types. Similarly, disease status (first-time treatment, recurrence, or secondary tumor) was unrelated to distress. The presence of further somatic disorders also had no influence on distress. In contrast, some other disease-related parameters showed associations with psychosocial distress. As shown in Fig. 2, a higher proportion of patients with metastases had high distress levels (*χ* ^2^ = 14.5, *df* = 1, *p* < 0.001). The same was true for participants who reported taking psychotropic substances (*χ* ^2^ = 18.2, *df* = 1, *p* < 0.001) or who had received psychological treatment in the past (*χ* ^2^ = 24.7, *df* = 1, *p* < 0.001). The patient's functional status also played an important role: The more limited patients were in their daily activities, the more likely they were to be rated as distressed.

Concerning the type of treatment, interestingly, having an operation was associated with lower distress levels. While 62.8% of the 239 patients without an operation showed high distress according to the PO-Bado SF, this was the case for only 48.6% of the 211 patients with an operation (*χ* ^2^ = 9.20, *df* = 1, *p* = 0.002). In contrast, chemotherapy was unrelated to distress. Radiotherapy and hormone therapy were associated with higher distress levels; however, only very small numbers of patients received these therapies (13 and nine, respectively).

### Combined influence of the significant predictors on psychosocial distress

A logistic regression was conducted to assess the combined impact of the identified significant predictors on PO-Bado SF distress ratings and to address the question which of the predictors could explain best whether a patient was distressed. The analysis showed that only four variables, namely, the psychological and physical distress factors (PO-Bado SF), the QSC-R23 score, and the fact that an operation was planned, had a significant impact on psychosocial distress (Table 4). The most influential variable for distress ratings was the psychological distress factor: For every additional point received on this factor, the probability of receiving a high distress rating was increased by a factor of 7.4 (odds ratio). The total explained variance of the model amounted to 68% (Nagelkerke’s *R* ^2^). The remaining variables, although showing significant relationships to distress ratings if tested separately, did not have a predictive value in addition to the variables mentioned above; 86.5% of the 222 patients were correctly classified on the basis of the logistic regression model.

**Table 4:** Logistic regression for the prediction of the PO-Bado SF distress rating (*N* = 222, excluding patients with missing values for the variable “metastases”, see the “Results” section for details)

	*b*	SE	Wald	Significance	Odds
Psychological distress	2.00	0.35	32.93	<0.01	7.40
Physical distress	0.72	0.33	4.94	0.03	2.06
QSC score	0.04	0.02	6.60	0.01	1.05
Age	−0.02	0.02	1.46	0.23	0.98
Gender	20.19	0.44	0.19	0.66	0.83
Metastases	0.29	0.47	0.39	0.53	1.34
Psychotropic medication	−0.94	0.53	3.18	0.08	0.39
Psychological treatment	−0.20	0.26	0.62	0.43	0.82
Actual functioning	0.19	0.50	0.06	0.81	1.13
Operation	−1.14	0.48	5.55	0.02	0.32
Cox and Snell *R* ^2^	0.509				
Nagelkerke’s *R* ^2^	0.680				

*b* parameter estimate (regression coefficient), *SE* estimated standard error, *Wald* Wald test, *Odds* odds ratio (exponent of *b*)

## Discussion

The present study investigated psychosocial distress in cancer patients in an acute care setting. A new expert rating tool, the PO-Bado short form and its breast cancer version [24], was used to determine cancer-related distress in a large group of patients. For validation purposes, this instrument was compared with a self-rating scale for stress in cancer patients [18]. In addition, we related sociodemographic, psychological, and disease-related factors to the PO-Bado SF to identify potential further predictors of psychosocial distress. All patients meeting the inclusion criteria were recruited, and only <1% of potential participants refused to take part in the investigation. This excludes a bias toward higher distress ratings that may be introduced by including only those patients who ask for psychological support or who are suspected to be distressed by doctors or nurses. The screenings were conducted as part of the clinical routine, demonstrating both the suitability of the PO-Bado SF for this purpose and the feasibility of a psycho-oncological assessment in an acute care setting.

According to the assessment with the PO-Bado SF, high levels of distress were diagnosed in 56.3% of the patients. Using the HADS [16], distress rates for new cancer patients were estimated at 26% [5]. Other studies have found similarly low levels only in patients with specific types of neoplasias like gastrointestinal [4] or gynecological cancers [27]. More common are reports of distress in 40–50% of patients [15, 27–29]. Higher levels (>60%) have been found when particular types of cancer were investigated [30]. The present results thus ranged at the higher end of the existing reports. Possibly, an external rating serves to identify a higher rate of distressed patients.

The known discrepancy between self-rating and external ratings of distress [12, 13] was observed also in the present study. According to the QSC-R23, only a bit more than half as many patients were classified as distressed compared with the PO-Bado SF. As there is no objective way of determining psychosocial distress, we may only speculate about the factors underlying this observation. It has been suggested that patients tend to underestimate their distress because of denial tendencies [21], which would be consistent with the lower stress rate obtained with the self-rating scale. On the other hand, external observers may overrate the patients’ distress [31]. However, both instruments yielded consistent results in nearly 70% of patients, and only very few patients who were classified as distressed with the QSC-R23 were not identified by the PO-Bado SF. In contrast, half of the patients who were identified as distressed by experts did not rate themselves as distressed. Future studies are required to shed more light on the causes underlying this discrepancy.

Previous studies on the role of sociodemographic factors have yielded heterogeneous results. In the present study, age was the only sociodemographic variable with a predictive value for psychosocial distress: Distressed patients were younger than nondistressed patients. Further sociodemographic factors like personal status variables, including partnership, presence of children, or employment status, were unrelated to psychosocial distress.

Patients who were classified as distressed were on average 3 years younger than patients without distress. This implies a rather limited clinical significance of the factor age. Findings in the recent literature concerning the influence of age have been heterogeneous, with only some investigations finding younger patients to be more distressed [24, 30, 32]. For example, a review by Aschenbrenner et al. [32] reported increased levels of anxiety in younger compared with older patients in eight out of 13 studies and distress in four out of 10 investigations. This tendency toward higher anxiety or distress in younger patients was interpreted as reflecting the increased prevalence of these disorders in younger individuals in the general population.

In line with the present study, the review by Aschenbrenner et al. [32] did not find evidence for an influence of gender on the prevalence of psychological distress or mental disorder. Herschbach et al. [26] reported higher physical distress in men and higher psychological distress in female patients. However, these effects could be attributed to differences in diagnoses and types of hospitals. The present findings are in contrast to some reports, suggesting higher distress rates in women than men [28, 33, 34]. For example, Goerling et al. [28] found that a higher proportion of female patients on a surgical ward were considered in need of psycho-oncological treatment. The authors interpreted this finding as reflecting an increased fear of operations in women and/or a higher tendency toward socially desirable responding in men. There is similarly little evidence for an influence of partnership status. The review cited above [32] found higher distress for singles compared with patients living with a partner in only two out of 15 studies. Future studies might focus more on the patients’ satisfaction with their partnerships.

Concerning disease-related variables, receiving surgical treatment was the most prominent factor associated with (lower) psychosocial distress. It was also the only external factor for which the regression analysis demonstrated a predictive value for distress in addition to the variables measured by the expert and self-rating scales. Receiving surgical treatment lowered the probability of receiving a high distress rating by one third. Lower distress scores for patients with surgery have also been reported by previous investigations [26, 28]. This may partly reflect the patients’ hope that the cancer may be removed and cured by this intervention. Conversely, a non-operable tumor typically represents a more advanced disease stage, which is experienced as more threatening. This was also reflected by the higher distress in patients with metastases or with more pronounced limitations of their daily activities.

Past psychological treatment was associated with higher distress. This finding is consistent with previous investigations showing increased distress levels in cancer patients with a history of psychiatric disease [24, 33–36]. It is conceivable that patients with a predisposition toward affective disorders react more strongly to a cancer diagnosis or may have reduced coping resources. On the other hand, those patients might also be more aware of psychosocial stress and be more prepared to express related feelings. Current psychotropic medication was also positively associated with distress [24, 34].

While we observed different distress levels in patients with different types of cancer, the relationship between cancer site and psychosocial distress did not reach significance. Variations in distress rates as a function of cancer type have been reported by previous studies [4, 27, 28]. Possibly in the present investigation, the number of patients with highly malignant tumors like pancreatic or bronchial cancer [27] was too low to identify associations between cancer type and distress level.

Both health service research and clinical experience have suggested that a sizeable proportion of cancer patients does not receive the psycho-oncological support they require [11, 37]. This is mainly attributable to an insufficient identification of these patients' need for treatment. Calls for routine screening have therefore been included in international and German cancer treatment guidelines [20, 38]. As screening without offering treatment would be unethical, we have offered each participant in our study a psycho-oncological counseling session. In general, we expect that an increasing awareness of psychological distress in cancer patients, as demonstrated in the present study, will lead to improved psycho-oncological treatment facilities.

## Conclusion

The present study adds to the existing evidence suggesting that a substantial proportion of cancer patients in acute care are psychosocially distressed. We successfully applied the PO-Bado SF [24], a short expert rating scale, to a large number of patients in an acute care environment. This demonstrates the feasibility of routine distress screenings in acute hospitals. Since a sizeable number of distressed patients may remain unrecognized, only such a systematic screening of patients shortly after admission would allow to offer psycho-oncological support to those who are most in need [12]. Expert and self-ratings with the QSC-R23 showed a good degree of correspondence, with the PO-Bado SF identifying a higher proportion of distressed patients than the self-rating instrument. Apart from younger age, the main factors associated with psychosocial distress were the presence of metastases, current psychotropic medication, and past psychological interventions. Having surgical treatment was associated with lower distress levels. Future studies may assess whether patients who are identified as highly distressed with the PO-Bado SF are actually willing to obtain psycho-oncological support and to which extent they benefit from it.
