# Correction to: Psychosocial distress in acute cancer patients assessed with an expert rating scale

**DOI:** 10.1007/s00520-020-05305-3

**Published:** 2020-02-27

**Authors:** Bianca Senf, Holger Brandt, Axel Dignass, Rolf Kleinschmidt, Jochen Kaiser

**Affiliations:** 1 Medical Clinic, Markus Hospital, Wilhelm-Epstein-Str. 2, 60431 Frankfurt am Main, Germany; 2 Institute of Medical Psychology, Goethe University, 60528 Frankfurt am Main, Germany; 3 Institute of Psychology, Goethe University, 60054 Frankfurt am Main, Germany

**Correction to: Support Care Cancer (2010) 18:957–965**

10.1007/s00520-010-0850-9

The article “Psychosocial distress in acute cancer patients assessed with an expert rating scale”, written by Bianca Senf, Holger Brandt, Axel Dignass, Rolf Kleinschmidt and Jochen Kaiser, was originally published electronically on the publisher’s internet portal (currently SpringerLink) on 11 April 2010 without open access.

With the author(s)’ decision to opt for Open Choice the copyright of the article changed on January 16, 2020 to © The Author(s) 2020 and the article is forthwith distributed under the terms of the Creative Commons Attribution 4.0 International License (https://creativecommons.org/licenses/by/4.0/), which permits use, sharing, adaptation, distribution and reproduction in any medium or format, as long as you give appropriate credit to the original author(s) and the source, provide a link to the Creative Commons license, and indicate if changes were made.

